# Dangling centrality highlights critical nodes by evaluating network stability through link removal

**DOI:** 10.1038/s41598-025-24930-8

**Published:** 2025-11-20

**Authors:** Ubaida Fatima, Saman Hina, Muhammad Wasif

**Affiliations:** 1 Department of Mathematics, NED University of Engineering and Technology, Karachi, Pakistan; 2 Department of Computing, Imperial College London, London, UK; 3 VelocityX, Karachi, Pakistan

**Keywords:** Centrality metrics, Social network analysis, Dangling centrality metric, Bitcoin dataset, Protein–protein interaction network, Mathematics and computing, Physics

## Abstract

This study introduces “Dangling Centrality,” a novel metric for identifying critical nodes in networks by assessing the impact of their link removal on system dynamics. The proposed metric is validated on real-world datasets, including Amazon product networks, a Protein–Protein Interaction (PPI) network, and a Bitcoin network, offering insights into key products, critical proteins, and influential entities. These nodes, as the main pillars of system propagation, are crucial for maintaining the structural and functional integrity of the network. By removing the links of these nodes, the network’s stability, flow, and communication can be disrupted, highlighting their importance. Additionally, small-scale 5-node and 6-node networks are analyzed to demonstrate the metric’s behavior in simpler contexts. Correlation analyses using Pearson’s, Spearman’s, and Kendall’s coefficients demonstrate alignment with traditional centrality metrics while providing a unique perspective. The findings emphasize the metric’s practical utility in understanding network vulnerabilities, enhancing resilience, and informing system design. Materials and implementations are available at: https://github.com/Ubaidafatima/Centrality-Measures.

## Introduction

The “Social Network Analysis (SNA)” is employed to understand the various connections among individuals, families, households, villages, communities, regions, and other social units. Social Networks are a structural configuration comprising individuals or groups referred to as "nodes," interconnected by various specific types of relationships^1–3^. Utilizing the findings from SNA can offer valuable insights into various domains such as human behavior, public health, organizational dynamics, and political science, thereby enhancing our comprehension of social systems and informing decision-making processes^4^. In^5^ the study addresses that gap by outlining the SNA process, comparing key tools and languages, and highlighting its applications across multidisciplinary areas.

The study in^6^ presents a comprehensive review of Social Network Analysis (SNA) techniques as applied to online social platforms, emphasizing the fundamentals of network representation, structural properties, and key analytical measures. It further explores modern developments in SNA applications such as influence modeling, link prediction, and information diffusion, while addressing growing concerns related to user privacy. By providing a comparative overview of current methodologies and highlighting open research challenges, this work aims to support future investigations and practical implementations of SNA in real-world digital environments.

Social Network Analysis (SNA) has seen the development of numerous centrality metrics, each aimed at identifying influential nodes within a network. While these metrics such as Degree Centrality, Closeness Centrality, and Eigenvector Centrality have proven effective in various contexts, they often face limitations in capturing the dynamic nature of real-world networks. For instance, traditional metrics primarily focus on connectivity or influence within the network but fail to address scenarios where the absence of critical entities disrupts communication. This gap becomes evident in domains such as business networks, biological systems, cryptocurrency ecosystems, and healthy lifestyle networks, where identifying nodes critical to maintaining seamless communication is paramount.

To address these deficiencies, Dangling Centrality is introduced, focusing on assessing a node’s importance by examining the impact of removing its connections or reducing its degree to zero. This method evaluates how the absence of a node link disrupts communication across the entire network, offering a distinct perspective for identifying and prioritizing key entities.

In many real-world networks, traditional centrality measures such as Degree Centrality (DC) and Betweenness Centrality (BC) focus on the presence and connectivity of nodes within the network. While these metrics effectively identify influential nodes based on their structural properties, they often fail to account for the criticality of nodes when their absence disrupts communication or network dynamics. For instance, in networks with bridge-type connections, the removal of a high-betweenness node may cause significant fragmentation. However, certain nodes that may not rank highly in traditional metrics could still play a pivotal role in maintaining overall network efficiency. This gap highlights the need for a metric like Dangling Centrality, which evaluates the importance of a node by simulating its removal and assessing the resulting impact on information flow. For example, in a Protein–Protein Interaction (PPI) network, removing a node identified by Dangling Centrality might reveal disruptions in key biological pathways that are overlooked by traditional measures. This novel approach offers a complementary perspective, enabling a deeper understanding of node criticality and network resilience.

### Inferring social networks from real-life datasets

The rapid growth of social networks has spurred interest in Social Network Analysis (SNA) for business intelligence. While theoretical advancements in data mining have been made, a gap remains in applying these techniques to real-world datasets. Addressing challenges like data acquisition, community structures, and network dynamics can unlock business applications^7^. In the same vein, previous research has applied SNA to biological datasets, showcasing its versatility in identifying key role players in any community or network graph^8,9^.

Previous studies in SNA have primarily focused on evaluating how individual nodes influence a network by calculating their importance through popular centrality metrics, community detection techniques, and maximal clique analysis^10^. However, less attention has been given to studying the impact of the absence of influential nodes within a network. This gap is critical for applications such as business planning, disease prevention, or promoting a healthy lifestyle, where the absence of key nodes can disrupt communication or cause system failure. To address this, the proposed method evaluates the effect of removing an influential node, referred to as a "*dangling node*," as detailed in Sect. 4.

### Contributions of this research

i. Proposed a novel Dangling Centrality approach for assessing link significance within intricate network frameworks in comparison to State-of-the-art (SOTA) conventional centrality measures.
ii. Complete evaluation against the baseline approaches that deal with the analysis of the presence of nodes in network datasets. In contrast, the proposed approach focuses on understanding how the removal of a vertex, node, person, protein, or customer can impact network communication dynamics. This presents a new method of determining node essentiality based on its communication absence, which can disrupt network communication.
iii. The method evaluates the impact of removing critical node links to enable proactive planning and prevent communication failures.

## Related work

Social Network Analysis (SNA) provides a framework to study the flow of resources, including information, among entities. Haythornthwaite^11^ emphasized the significance of analyzing these exchange patterns, where actors act as nodes and relationships represent connections, to improve information delivery and control mechanisms. Newman and Girvan^12^ introduced algorithms for community detection through iterative link elimination and stability analysis, laying foundational methods for analyzing network configurations. Houghton^13^ highlighted the role of SNA in command and control during emergency services, focusing on network structure and information flow.

Fioriti and Marta^14^ proposed a spectral method to identify sources of disease outbreaks within contact networks, demonstrating the application of centrality measures in epidemic analysis. Fiz et al.^15^ introduced "mint centrality," a novel metric tailored for Bitcoin transaction networks, emphasizing the importance of customized centrality metrics in unique contexts. Saqr and Alamro^16^ explored SNA in online problem-based learning, showing how centrality measures can reveal the roles of participants in educational interactions.

Atsalakis et al.^17^ demonstrated the predictive power of a hybrid neuro-fuzzy model for Bitcoin price trends, while^18^ introduced “Isolating Centrality” to detect critical nodes in complex networks, outperforming traditional centrality measures. Fatima et al.^8^ proposed the global clustering coefficient-dependent degree centrality (GCCDC) metric, which addressed limitations of existing measures and provided insights into protein–protein interaction networks.

Nasiri et al.^19^ developed Weighted Common Neighbors (WCN), a link prediction method integrating centrality measures, highlighting the role of interlayer information in multiplex networks. Zhao and Sun^20^ introduced weighted Laplacian energy centrality to identify influential nodes in aviation networks, demonstrating the metric’s effectiveness in maintaining network robustness.^21^ reviewed advancements in machine learning for biological networks, underscoring centrality measures’ role in drug interaction prediction and gene identification.

Applications of SNA extend beyond centrality. Hung et al.^22^ analyzed sentiment and social network connections on COVID-19-related tweets, while^23^ leveraged topological and biological features for SARS-CoV-2 gene identification. Rostami et al.^24^ reviewed the application of community detection in healthcare datasets, identifying challenges and knowledge gaps in this domain.

Also,^7^ addressed the ambiguity in protein interaction strength in PPI networks by introducing Bio-Link Strength, a fuzzy membership function that quantifies interactions on a continuous scale. They extended traditional centrality measures (degree, closeness, betweenness, eigenvector) to fuzzy measures (e.g., fuzzy connectivity, fuzzy influence centrality) and demonstrated the framework’s scalability across multiple PPI datasets. Results highlighted the superior performance of fuzzy measures, particularly fuzzy connectivity and influence centrality, in identifying crucial proteins, validated through Gene Ontology analysis and correlation studies.

While classical Social Network Analysis (SNA) has been widely applied across various domains—ranging from sociology to computer science—emerging technologies are now reshaping its scope and capabilities. One such advancement is the integration of quantum computing with SNA, giving rise to Quantum Social Network Analysis (QSNA). With the integration of quantum computing into network science, QSNA has recently emerged as a promising paradigm, offering novel approaches to classical SNA tasks through quantum-enhanced algorithms, while addressing the complexity of large-scale social systems^23^.

These studies collectively illustrate the evolution and significance of centrality measures and network analysis in understanding complex systems, from healthcare and finance to online learning and digital currencies. The proposed work builds upon this foundation by addressing the limitations of existing centrality metrics and introducing new measures tailored for large, real-world datasets.

## Utilization of Prominent centrality metrics in the analysis of networks

As per findings presented by various researchers in the literature, the identification of key vertices in a social network graph that is $G(V,E)$, where $V$ demonstrates nodes/vertices and $E$ demonstrates edges/links can be achieved through the calculation of centrality metrics^26–29^. The following sub-sections provide the mathematical formulation of various centrality measures.

### Degree centrality metric (DC)

This measure of a node can be recognized by capturing the incoming and outgoing connections of a node^1,8,30,31^. Computation of the degree centrality metric can be easily computed by the formation of an ***“Adjacency Matrix*** $({{\varvec{A}}}_{{\varvec{g}}})"$, which is represented in this section by two considered small network graphs and demonstrated as ${{\varvec{A}}}_{{\varvec{g}}1}$ **for 5-nodes graph** and ${{\varvec{A}}}_{{\varvec{g}}2}$ **for 6-nodes graph** in Eqs. (1) and (2) respectively.

1 $${{\varvec{A}}}_{{\varvec{g}}1}= \left[\begin{array}{ccc}0& 1& \begin{array}{ccc}1& 1& 0\end{array}\\ 1& 0& \begin{array}{ccc}1& 0& 0\end{array}\\ \begin{array}{c}1\\ 1\\ 0\end{array}& \begin{array}{c}1\\ 0\\ 0\end{array}& \begin{array}{ccc}\begin{array}{c}0\\ 0\\ 0\end{array}& \begin{array}{c}0\\ 0\\ 1\end{array}& \begin{array}{c}0\\ 1\\ 0\end{array}\end{array}\end{array}\right]$$

2 $${{\varvec{A}}}_{{\varvec{g}}2}=\left[\begin{array}{ccc}0& 1& \begin{array}{ccc}0& 0& \begin{array}{cc}1& 0\end{array}\end{array}\\ 1& 0& \begin{array}{ccc}1& 1& \begin{array}{cc}1& 0\end{array}\end{array}\\ \begin{array}{c}0\\ 0\\ \begin{array}{c}1\\ 0\end{array}\end{array}& \begin{array}{c}1\\ 1\\ \begin{array}{c}1\\ 0\end{array}\end{array}& \begin{array}{c}\begin{array}{ccc}0& 1& \begin{array}{cc}0& 1\end{array}\end{array}\\ \begin{array}{ccc}1& 0& \begin{array}{cc}1& 1\end{array}\end{array}\\ \begin{array}{c}\begin{array}{ccc}0& 1& \begin{array}{cc}0& 1\end{array}\end{array}\\ \begin{array}{ccc}1& 1& \begin{array}{cc}1& 0\end{array}\end{array}\end{array}\end{array}\end{array}\right]$$

**Fig. 1:**
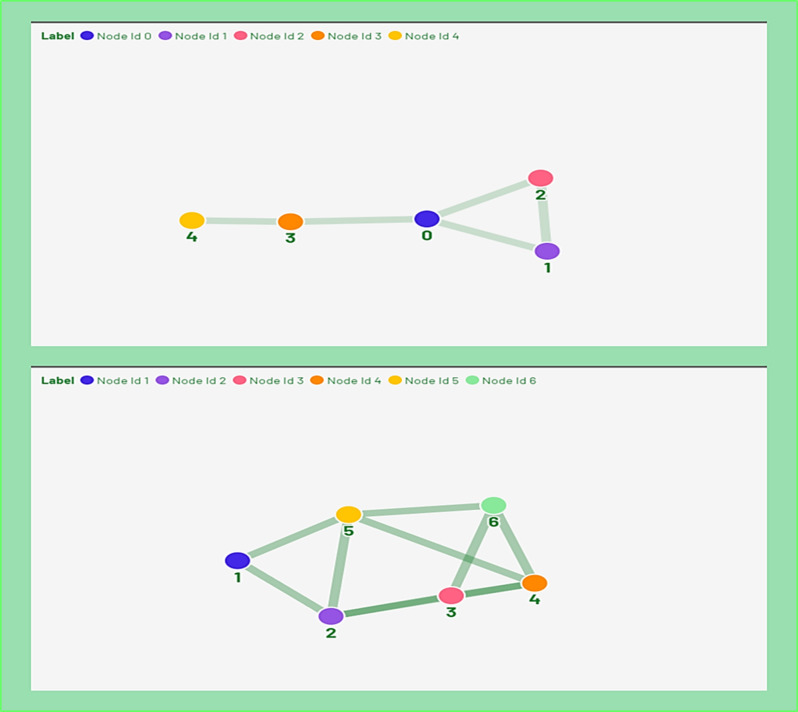
Undirected simple networks: **a** of (5 nodes, 5 edges) and **b** of (6 nodes, 9 edges).

This metric proves valuable in identifying the popularity of nodes and assessing their influence based on the degree of connectivity. Figure 1a, b clearly demonstrate which node is highly connected and which node has minimum connections. For instance, in Fig. 1b Node ID 5 has a maximum degree (i.e. 4) and Node ID 1 has a minimum degree (i.e. 2) shown in Table 1.

**Table 1:** Degree Centrality (DC) Computation for Fig. 1b.

Node ID	1	2	3	4	5	6
DC	2	3	3	3	4	3

### Betweenness centrality (BC)

To compute the Betweenness Centrality (BC) for a specific node ${\varvec{u}}$, identify all shortest paths between nodes $i$ and $j$ that pass through ${\varvec{u}}$. The centrality value for u is then obtained by dividing the number of such paths by the total number of shortest paths between $i$ and $j$ in the network^8,33^. Product with high betweenness measures captures that product (node) plays a vital role in the sale of other products (nodes) in the product network^34^. For instance, computation of betweenness centrality (BC) for nodes in Fig. 1b is shown in Table 2.

**Table 2:** Betweenness Centrality (BC) Computation for a graph in Fig. 1b.

*For Node ID 1:* **The BWC for node 1 is 0**	*For Node ID 4:* $[\text{2,6}]\to 1/3$ $[\text{3,5}]\to 1/3$ $[\text{5,3}]\to 1/3$ $\left[\text{6,2}\right]\to 1/3$. The sum of all sets is **the BWC of node 4 i.e. 1.3333**
*For Node ID 2:* $[\text{1,3}]\to 1/1$ $[\text{1,4}]\to 1/2$ $\left[\text{3,1}\right]\to 1/1$ $[\text{3,5}]\to 1/3$ $[\text{4,1}]\to 1/2$ $[\text{5,3}]\to 1/3$ The sum of all sets is the **BWC of node 2 i.e. 3.6667**	*For Node ID 5:* $[\text{1,4}]\to 1/2$ $[\text{1,6}]\to 1/1$ $[\text{2,6}]\to 1/3$ $[\text{4,1}]\to 1/2$ $[\text{6,1}]\to 1/1$ $\left[\text{6,2}\right]\to 1/3$ The sum of all sets is the **BWC of node 5 i.e. 3.6667**
*For Node ID 3* *:* $[\text{2,6}]\to 1/3$ $[\text{6,2}]\to 1/3$ The sum of all sets is the **BWC of node 3 i.e. 0.6667**	*For Node ID 6:* $\left[\text{3,5}\right]\to 1/3$ $\left[\text{5,3}\right]\to 1/3$. The sum of all sets is the **BWC of node 6 i.e. 0.6667**

### Closeness centrality (CC)

The Closeness Centrality (CC) of a node is determined by taking the reciprocal of the sum of the shortest paths from that node to all other nodes in the network^8,35^.

Therefore, the “**Shortest Path Distance Matrix (SPDM)**” was evaluated using Eq. (3) which is used to calculate the closeness centrality measure for every node in Fig. 1b. Node values were calculated using Eq. (3) which is tabulated in Table 3. These values clearly show that **Node ID 2, Node ID 4 and Node ID 5** are the most central nodes. For PPI data, when protein has high ‘**CC**’ means it is more central protein in the considered yeast network.

**Table 3:** Closeness Centrality (CC) measure for a simple 6 node network in Fig. 1b.

Node ID	1	2	3	4	5	6
CC	0.1250	0.1667	0.1428	0.1667	0.1667	0.1428

3 $$\begin{aligned} SPDM & = \left[ {\begin{array}{*{20}l} 0 \hfill & 1 \hfill & 2 \hfill & 2 \hfill & 1 \hfill & 2 \hfill \\ 1 \hfill & 0 \hfill & 1 \hfill & 1 \hfill & 1 \hfill & 2 \hfill \\ 2 \hfill & 1 \hfill & 0 \hfill & 1 \hfill & 2 \hfill & 1 \hfill \\ 2 \hfill & 1 \hfill & 1 \hfill & 0 \hfill & 1 \hfill & 1 \hfill \\ 1 \hfill & 1 \hfill & 2 \hfill & 1 \hfill & 0 \hfill & 1 \hfill \\ 2 \hfill & 2 \hfill & 1 \hfill & 1 \hfill & 1 \hfill & 0 \hfill \\ \end{array} } \right] \Rightarrow \sum S PDM = \left[ {\begin{array}{*{20}l} 8 \hfill \\ 6 \hfill \\ 7 \hfill \\ 6 \hfill \\ 6 \hfill \\ 7 \hfill \\ \end{array} } \right] \\ & \Rightarrow CC(i) = \frac{1}{{\sum S PDM}} = \left[ {\begin{array}{*{20}l} {0.1250} \hfill \\ {0.1667} \hfill \\ {0.1428} \hfill \\ {0.1667} \hfill \\ {0.1667} \hfill \\ {0.1428} \hfill \\ \end{array} } \right] \\ \end{aligned}$$

### Eigenvector centrality (EVC)

The EigenVector Centrality (EVC) is used to quantify a node’s significance by focusing on its linked nodes. A node that is surrounded by highly linked nodes and has number of links, has the highest EVC^8,35,36^.

The ${\varvec{n}}$ number of EigenValues are computed and their corresponding eigenvectors from ${\varvec{n}}\times {\varvec{n}}$ size of Adjacency matrix ${A}_{g}.$ EigenVector Centrality (EVC) is computed by applying “Power method” to ***Adjacency Matrix ***$({{\varvec{A}}}_{{\varvec{g}}})$***.*** For instance, Fig. 1b presented 6 undirected nodes network graph, where Node ID 2, Node ID 4 and Node ID 5 are connected to highly linked nodes, therefore, it must have highest EVC as computed in Table 4.

**Table 4:** Evaluation of EigenVector Centrality (EVC) for Eq. (2).

$$\begin{aligned} EV1 & = \left[ {\begin{array}{*{20}l} 0 \hfill & 1 \hfill & 0 \hfill & 0 \hfill & 1 \hfill & 0 \hfill \\ 1 \hfill & 0 \hfill & 1 \hfill & 1 \hfill & 1 \hfill & 0 \hfill \\ 0 \hfill & 1 \hfill & 0 \hfill & 1 \hfill & 0 \hfill & 1 \hfill \\ 0 \hfill & 1 \hfill & 1 \hfill & 0 \hfill & 1 \hfill & 1 \hfill \\ 1 \hfill & 1 \hfill & 0 \hfill & 1 \hfill & 0 \hfill & 1 \hfill \\ 0 \hfill & 0 \hfill & 1 \hfill & 1 \hfill & 1 \hfill & 0 \hfill \\ \end{array} } \right]\left[ {\begin{array}{*{20}l} 1 \hfill \\ 1 \hfill \\ 1 \hfill \\ 1 \hfill \\ 1 \hfill \\ 1 \hfill \\ \end{array} } \right] \\ & = \left[ {\begin{array}{*{20}l} 2 \hfill \\ 4 \hfill \\ 3 \hfill \\ 4 \hfill \\ 4 \hfill \\ 3 \hfill \\ \end{array} } \right] \\ \end{aligned}$$ $$\begin{aligned} Normalized value &=n1 \\ &=\sqrt{{2}^{2}+{4}^{2}+{3}^{2}+{4}^{2}+{4}^{2}+{3}^{2}}\\ & =8.3666 \end{aligned}$$ $$\begin{aligned} EVC1 & = \frac{{EV1}}{{n1}} = \left[ {\begin{array}{*{20}l} {0.2390} \hfill \\ {0.4781} \hfill \\ {0.3586} \hfill \\ {0.4781} \hfill \\ {0.4781} \hfill \\ {0.3586} \hfill \\ \end{array} } \right] \\ & \to Iteration\# 1 \end{aligned}$$	$$\begin{aligned} EV3 & = \left[ {\begin{array}{*{20}l} 0 \hfill & 1 \hfill & 0 \hfill & 0 \hfill & 1 \hfill & 0 \hfill \\ 1 \hfill & 0 \hfill & 1 \hfill & 1 \hfill & 1 \hfill & 0 \hfill \\ 0 \hfill & 1 \hfill & 0 \hfill & 1 \hfill & 0 \hfill & 1 \hfill \\ 0 \hfill & 1 \hfill & 1 \hfill & 0 \hfill & 1 \hfill & 1 \hfill \\ 1 \hfill & 1 \hfill & 0 \hfill & 1 \hfill & 0 \hfill & 1 \hfill \\ 0 \hfill & 0 \hfill & 1 \hfill & 1 \hfill & 1 \hfill & 0 \hfill \\ \end{array} } \right]\left[ {\begin{array}{*{20}l} {0.2760} \hfill \\ {0.4485} \hfill \\ {0.3795} \hfill \\ {0.4830} \hfill \\ {0.4485} \hfill \\ {0.3795} \hfill \\ \end{array} } \right] \\ & = \left[ {\begin{array}{*{20}l} {0.8971} \hfill \\ {1.5872} \hfill \\ {1.3111} \hfill \\ {1.6562} \hfill \\ {1.5872} \hfill \\ {1.3111} \hfill \\ \end{array} } \right] \\ \end{aligned}$$ $$\begin{aligned} n3 & = \sqrt {\begin{array}{*{20}l} {0.8971^{2} + 1.5872^{2} + 1.3111^{2} } \\ { + 1.6562^{2} + 1.5872^{2} + 1.3111^{2} } \\ \end{array} } \\ & = 3.4675 \\ \end{aligned}$$ $$\begin{aligned} EVC3 & = \frac{{EV3}}{{n3}} = \left[ {\begin{array}{*{20}l} {0.2587} \hfill \\ {0.4577} \hfill \\ {0.3781} \hfill \\ {0.4776} \hfill \\ {0.4577} \hfill \\ {0.3781} \hfill \\ \end{array} } \right] \\ & \to Iteration\# 3 \end{aligned}$$
$$\begin{aligned} EV2 & = \left[ {\begin{array}{*{20}l} 0 \hfill & 1 \hfill & 0 \hfill & 0 \hfill & 1 \hfill & 0 \hfill \\ 1 \hfill & 0 \hfill & 1 \hfill & 1 \hfill & 1 \hfill & 0 \hfill \\ 0 \hfill & 1 \hfill & 0 \hfill & 1 \hfill & 0 \hfill & 1 \hfill \\ 0 \hfill & 1 \hfill & 1 \hfill & 0 \hfill & 1 \hfill & 1 \hfill \\ 1 \hfill & 1 \hfill & 0 \hfill & 1 \hfill & 0 \hfill & 1 \hfill \\ 0 \hfill & 0 \hfill & 1 \hfill & 1 \hfill & 1 \hfill & 0 \hfill \\ \end{array} } \right]\left[ {\begin{array}{*{20}l} {0.2390} \hfill \\ {0.4781} \hfill \\ {0.3586} \hfill \\ {0.4781} \hfill \\ {0.4781} \hfill \\ {0.3586} \hfill \\ \end{array} } \right] \\ & = \left[ {\begin{array}{*{20}l} {0.9562} \hfill \\ {1.5538} \hfill \\ {1.3148} \hfill \\ {1.6733} \hfill \\ {1.5538} \hfill \\ {1.3148} \hfill \\ \end{array} } \right] \\ \end{aligned}$$ $$$$ \begin{aligned} n2 & = \sqrt {\begin{array}{*{20}l} {0.9562^{2} + 1.5538^{2} + 1.3148^{2} } \\ { + 1.6733^{2} + 1.5538^{2} + 1.3148^{2} } \\ \end{array} } \\ & = 3.4641 \\ \end{aligned} $$$$ $$\begin{aligned} EVC2 &= \frac{{EV2}}{{n2}} = \left[ {\begin{array}{*{20}l} {0.2760} \hfill \\ {0.4485} \hfill \\ {0.3795} \hfill \\ {0.4830} \hfill \\ {0.4485} \hfill \\ {0.3795} \hfill \\ \end{array} } \right] \\ &\to Iteration\# 2 \end{aligned}$$	$$\begin{aligned} EV4 & = \left[ {\begin{array}{*{20}l} 0 \hfill & 1 \hfill & 0 \hfill & 0 \hfill & 1 \hfill & 0 \hfill \\ 1 \hfill & 0 \hfill & 1 \hfill & 1 \hfill & 1 \hfill & 0 \hfill \\ 0 \hfill & 1 \hfill & 0 \hfill & 1 \hfill & 0 \hfill & 1 \hfill \\ 0 \hfill & 1 \hfill & 1 \hfill & 0 \hfill & 1 \hfill & 1 \hfill \\ 1 \hfill & 1 \hfill & 0 \hfill & 1 \hfill & 0 \hfill & 1 \hfill \\ 0 \hfill & 0 \hfill & 1 \hfill & 1 \hfill & 1 \hfill & 0 \hfill \\ \end{array} } \right]\left[ {\begin{array}{*{20}c} {0.2587} \\ {0.4577} \\ {0.3781} \\ {0.4776} \\ {0.4577} \\ {0.3781} \\ \end{array} } \right] \\ & = \left[ {\begin{array}{*{20}c} {0.9154} \\ {1.5722} \\ {1.3134} \\ {1.6717} \\ {1.5722} \\ {1.3134} \\ \end{array} } \right] \\ \end{aligned}$$ $$\begin{aligned} n4 & = \sqrt {\begin{array}{*{20}l} {0.9154^{2} + 1.5722^{2} + 1.3134^{2} } \\ { + 1.6717^{2} + 1.5722^{2} + 1.3134^{2} } \\ \end{array} } \\ & = 3.4679 \\ \end{aligned} $$$$ $$\begin{aligned} EVC4 &= \frac{{EV4}}{{n4}} = \left[ {\begin{array}{*{20}l} {0.2640} \hfill \\ {0.4533} \hfill \\ {0.3787} \hfill \\ {0.4820} \hfill \\ {0.4533} \hfill \\ {0.3787} \hfill \\ \end{array} } \right] \\ & \to Iteration\# 4 \end{aligned}$$
$$\begin{aligned} EV5 & = \left[ {\begin{array}{*{20}l} 0 \hfill & 1 \hfill & 0 \hfill & 0 \hfill & 1 \hfill & 0 \hfill \\ 1 \hfill & 0 \hfill & 1 \hfill & 1 \hfill & 1 \hfill & 0 \hfill \\ 0 \hfill & 1 \hfill & 0 \hfill & 1 \hfill & 0 \hfill & 1 \hfill \\ 0 \hfill & 1 \hfill & 1 \hfill & 0 \hfill & 1 \hfill & 1 \hfill \\ 1 \hfill & 1 \hfill & 0 \hfill & 1 \hfill & 0 \hfill & 1 \hfill \\ 0 \hfill & 0 \hfill & 1 \hfill & 1 \hfill & 1 \hfill & 0 \hfill \\ \end{array} } \right]\left[ {\begin{array}{*{20}l} {0.2640} \hfill \\ {0.4533} \hfill \\ {0.3787} \hfill \\ {0.4820} \hfill \\ {0.4533} \hfill \\ {0.3787} \hfill \\ \end{array} } \right] \\ & = \left[ {\begin{array}{*{20}l} {0.9067} \hfill \\ {1.5781} \hfill \\ {1.3141} \hfill \\ {1.6642} \hfill \\ {1.5781} \hfill \\ {1.3141} \hfill \\ \end{array} } \right] \\ \end{aligned}$$ $$\begin{aligned} n5 =\sqrt{{0.9067}^{2}+{1.5781}^{2}+{1.3141}^{2}+{1.6642}^{2}+{1.5781}^{2}+{1.3141}^{2}}\\ =3.4679 \to \lambda (Principal Eigenvalue) \end{aligned}$$ $$\begin{aligned} EVC5 = \frac{{EV5}}{{n5}} = \left[ {\begin{array}{*{20}l} {0.2615} \hfill \\ {0.4551} \hfill \\ {0.3789} \hfill \\ {0.4799} \hfill \\ {0.4551} \hfill \\ {0.3789} \hfill \\ \end{array} } \right] \\ \to Iteration\# 5 \end {aligned}$$	

### Katz centrality (KC)

Katz centrality (KC) is a metric specifically designed for directed networks^8,37,38^. As in network analysis, the amount of centrality measure for a node is not considered but greater or lesser centrality measured is taking into account. The Katz centrality (KC) for any node (product) can be calculated using Eq. (4).

4 $${C}_{katz}\left({v}_{i}\right)=\alpha \sum_{j=1}^{n}{A}_{j,i}{C}_{katz}\left({v}_{i}\right)+\beta$$

The first part of Eq. (4) is supervised by parameter $\boldsymbol{\alpha }$ and is pretty much same to eigenvector centrality and ${\mathbb{l}}$ is a unity column matrix. The second part contains the bias term ${\varvec{\beta}}$ that avoids the zero centrality measure. Therefore, for any node ${{\varvec{v}}}_{{\varvec{i}}}$ Katz centrality (KC) can be computed simplifying Eq. (4). The simplification is represented from Eqs. (5–8).

5 $${C}_{katz}=\alpha {A}^{T}{C}_{katz}+\beta .{\mathbb{l}}$$

6 $${C}_{katz}-\alpha {A}^{T}{C}_{katz}=\beta .{\mathbb{l}}$$

7 $${C}_{katz}\left(1- \alpha {A}^{T}\right)=\beta .{\mathbb{l}}$$

8 $${C}_{katz}=\beta {\left(1-\alpha {A}^{T}\right)}^{-1}.{\mathbb{l}}$$

As matrix is inverting, parameter $\boldsymbol{\alpha }$ cannot be supervised first part of Eq. (4) for all values. Computation for a network in Fig. 1b is mentioned in Table 5.

- If $\boldsymbol{\alpha }=0,$ EigenVector centrality part of Eq. (4) is removed and there will be the same Katz centrality (KC)” value ${\varvec{\beta}}$ for all nodes present in the network.
- If $\boldsymbol{\alpha }$ is higher, the influence of ${\varvec{\beta}}$ is decreased.
- If $$\boldsymbol{\alpha }=\frac{1}{{\varvec{\lambda}}}\boldsymbol{ }\left(where{\varvec{\lambda}}, largest eigenvalue centrality of {{\varvec{A}}}^{{\varvec{T}}}\right)$$ is taken, then the
- $\left|1-\boldsymbol{\alpha }{{\varvec{A}}}^{{\varvec{T}}}\right|=0$ that is matrix $1-\boldsymbol{\alpha }{{\varvec{A}}}^{{\varvec{T}}}$ becomes non-invertible that creates uncertainty in computation of KC or KC diverges. Generally, $\boldsymbol{\alpha }<\frac{1}{{\varvec{\lambda}}}$ is a selection criteria for computation of KC properly to attain fast convergence.

As $\lambda = 3.4679$ is obtained in Table 4 ,assuming $\alpha =0.2 {\text{and}} \beta =1$

**Table 5:** Evaluation of Katz Centrality for Fig. 2b.

$$\begin{aligned} C_{{katz}} & = 1*\left( {\left[ {\begin{array}{*{20}l} 1 \hfill & 0 \hfill & 0 \hfill & 0 \hfill & 0 \hfill & 0 \hfill \\ 0 \hfill & 1 \hfill & 0 \hfill & 0 \hfill & 0 \hfill & 0 \hfill \\ 0 \hfill & 0 \hfill & 1 \hfill & 0 \hfill & 0 \hfill & 0 \hfill \\ 0 \hfill & 0 \hfill & 0 \hfill & 1 \hfill & 0 \hfill & 0 \hfill \\ 0 \hfill & 0 \hfill & 0 \hfill & 0 \hfill & 1 \hfill & 0 \hfill \\ 0 \hfill & 0 \hfill & 0 \hfill & 0 \hfill & 0 \hfill & 1 \hfill \\ \end{array} } \right]} \right. \\ & \quad - 0.2{\text{*}}\left. {\left[ {\begin{array}{*{20}l} 0 \hfill & 1 \hfill & 0 \hfill & 0 \hfill & 1 \hfill & 0 \hfill \\ 1 \hfill & 0 \hfill & 1 \hfill & 1 \hfill & 1 \hfill & 0 \hfill \\ 0 \hfill & 1 \hfill & 0 \hfill & 1 \hfill & 0 \hfill & 1 \hfill \\ 0 \hfill & 1 \hfill & 1 \hfill & 0 \hfill & 1 \hfill & 1 \hfill \\ 1 \hfill & 1 \hfill & 0 \hfill & 1 \hfill & 0 \hfill & 1 \hfill \\ 0 \hfill & 0 \hfill & 1 \hfill & 1 \hfill & 1 \hfill & 0 \hfill \\ \end{array} } \right]} \right)^{{ - 1}} *\left[ {\begin{array}{*{20}l} 1 \hfill \\ 1 \hfill \\ 1 \hfill \\ 1 \hfill \\ 1 \hfill \\ 1 \hfill \\ \end{array} } \right] \\ \end{aligned}$$ $$\begin{aligned} C_{{katz}} & = \left( {\left[ {\begin{array}{*{20}l} 1 \hfill & 0 \hfill & 0 \hfill & 0 \hfill & 0 \hfill & 0 \hfill \\ 0 \hfill & 1 \hfill & 0 \hfill & 0 \hfill & 0 \hfill & 0 \hfill \\ 0 \hfill & 0 \hfill & 1 \hfill & 0 \hfill & 0 \hfill & 0 \hfill \\ 0 \hfill & 0 \hfill & 0 \hfill & 1 \hfill & 0 \hfill & 0 \hfill \\ 0 \hfill & 0 \hfill & 0 \hfill & 0 \hfill & 1 \hfill & 0 \hfill \\ 0 \hfill & 0 \hfill & 0 \hfill & 0 \hfill & 0 \hfill & 1 \hfill \\ \end{array} } \right]} \right. \\ & \quad - \left. {\left[ {\begin{array}{*{20}l} {0.0} \hfill & {0.2} \hfill & {0.0} \hfill & {0.0} \hfill & {0.2} \hfill & {0.0} \hfill \\ {0.2} \hfill & {0.0} \hfill & {0.2} \hfill & {0.2} \hfill & {0.2} \hfill & {0.0} \hfill \\ {0.0} \hfill & {0.2} \hfill & {0.0} \hfill & {0.2} \hfill & {0.0} \hfill & {0.2} \hfill \\ {0.0} \hfill & {0.2} \hfill & {0.2} \hfill & {0.0} \hfill & {0.2} \hfill & {0.2} \hfill \\ {0.2} \hfill & {0.2} \hfill & {0.0} \hfill & {0.2} \hfill & {0.0} \hfill & {0.2} \hfill \\ {0.0} \hfill & {0.0} \hfill & {0.2} \hfill & {0.2} \hfill & {0.2} \hfill & 0 \hfill \\ \end{array} } \right]} \right)^{{ - 1}} *\left[ {\begin{array}{*{20}l} 1 \hfill \\ 1 \hfill \\ 1 \hfill \\ 1 \hfill \\ 1 \hfill \\ 1 \hfill \\ \end{array} } \right] \\ \end{aligned}$$ $$\begin{aligned} C_{{katz}} & = \left( {\left[ {\begin{array}{*{20}l} {1.00} \hfill & { - 0.2} \hfill & { - 0.2} \hfill & { - 0.2} \hfill & {0.00} \hfill \\ { - 0.2} \hfill & {1.00} \hfill & { - 0.2} \hfill & {0.00} \hfill & {0.00} \hfill \\ { - 0.2} \hfill & { - 0.2} \hfill & {1.00} \hfill & {0.00} \hfill & {0.00} \hfill \\ { - 0.2} \hfill & {0.00} \hfill & {0.00} \hfill & {1.00} \hfill & { - 0.2} \hfill \\ {0.00} \hfill & {0.00} \hfill & {0.00} \hfill & { - 0.2} \hfill & {1.00} \hfill \\ \end{array} } \right]} \right)^{{ - 1}} *\left[ {\begin{array}{*{20}l} 1 \hfill \\ 1 \hfill \\ 1 \hfill \\ 1 \hfill \\ 1 \hfill \\ 1 \hfill \\ \end{array} } \right] \\ & \quad = \left[ {\begin{array}{*{20}l} {2.4059} \hfill \\ {3.5146} \hfill \\ {3.0335} \hfill \\ {3.6192} \hfill \\ {3.5146} \hfill \\ {3.0335} \hfill \\ \end{array} } \right] \\ \end{aligned}$$	

**Fig. 2:**
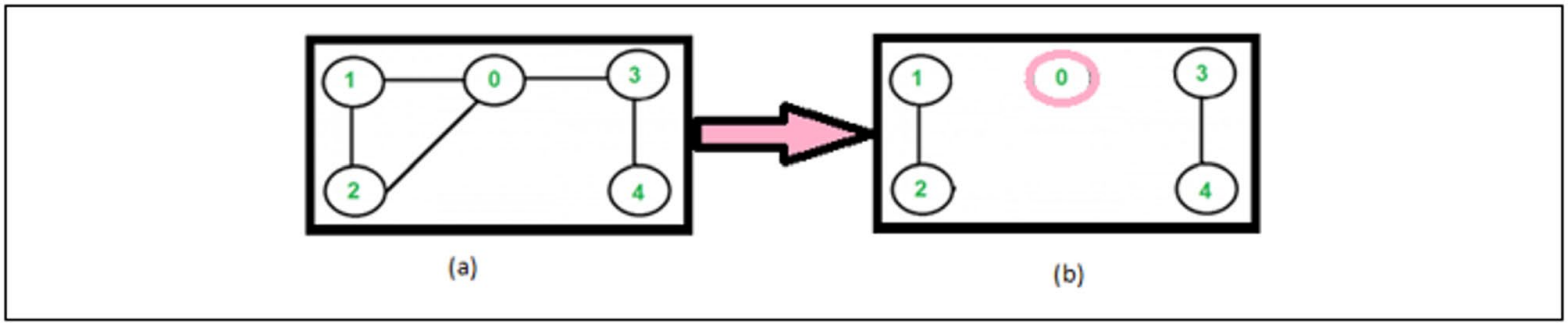
Illustration of the dangling centrality evaluation concept.

Table 6 presents a comprehensive comparison of the six centrality metrics. The analysis reveals the level of influence associated with specific Node IDs within the graphs depicted in Fig. 1a, b. It highlights the identification of nodes that play a more pivotal role and examines the dependencies of other nodes on these influential ones for effective communication within the network.

**Table 6:** Dangling Centrality measure for a small undirected graph of 5 nodes.

Node ID $({\varvec{i}})$	0	1	2	3	4
${\boldsymbol{\varnothing }}_{{\varvec{c}}}({\varvec{i}})$	0.72093	0.3953	0.3953	0.58139	0.30232

Nodes with **IDs 2, 4, and 5** consistently exhibit the highest values across all centrality metrics, signifying their role as the most influential entities in the simple 6-node network responsible for information transfer and communication among other members. In the realm of marketing and product network analysis, the significance of Node IDs **2, 4, and 5** can be elucidated by understanding that the prominent presence of these nodes (representing products) in a store has the potential to enhance the market value of interconnected neighbors (other products).

In the next section, a groundbreaking technique for identifying influential actors within a complex network dataset is introduced. To comprehensively assess its efficacy, **Dangling Centrality** $({{\varvec{\phi}}}_{{\varvec{c}}})$ is compared with the established centrality metrics discussed in the preceding section.

## Dangling centrality $({{\varvec{\phi}}}_{{\varvec{c}}})$: a novel metric

The major task in handling graph networks is to determine the key factors that efficiently deliver information within the vertices. The novel **Dangling Centrality** $({{\varvec{\phi}}}_{{\varvec{c}}})$ provides a framework to quantify the power of each node in a network by deleting its communication with other nodes or, more specifically, by removing its edges (links). This approach determines the prominence of each node in conveying information throughout the network. The importance of a node is assessed by evaluating its Dangling Centrality, which measures the impact on the network’s communication or configuration when the node’s degree is reduced to zero, highlighting how its links absence affects the flow of information within the network. The higher the value of Dangling Centrality $({\phi }_{c})$, the higher will be its role in the network organization and information delivering.

The term “Dangling” draws attention to the “PageRank” concept, which suggests that a web page with "no outgoing links" can be likened to a node that lacks any connections^39–41^. These studies describe such web pages as nodes that do not direct to any other node or web page, referring to them as "Dangling nodes." Building on this idea, I have adopted the term “Dangling” to develop a centrality measure aimed at addressing the complexities within Social Network Analysis (SNA) studies.

Termed **Dangling Centrality **$({{\varvec{\phi}}}_{{\varvec{c}}})$**,** this novel metric adopts a unique perspective. Unlike traditional centrality metrics, which assess the significance of a node based on its connections and communication presence, **Dangling Centrality** $\left({{\varvec{\phi}}}_{{\varvec{c}}}\right)$ takes a distinctive approach by evaluating the consequences of a node links absence in the network. This innovative metric provides insights into how network communication dynamics are affected when a specific node is removed, offering a nuanced understanding of node importance in the context of network structure and robustness.

Also, ${{\varvec{\phi}}}_{{\varvec{c}}}({\varvec{G}})$ uses the Shortest Path Distance Matrix (SPDM) to measure the impact of isolated nodes after their edges are removed. When edges are absent, the SPDM highlights how these isolated nodes affect the flow of information, with their centrality reflecting the disruption they cause to network connectivity. ${{\varvec{\phi}}}_{{\varvec{c}}}({\varvec{G}})$ of a network graph can be measured through Eq. (9) and Eq. (10). Excluding '0' entries in the Shortest-Path Distance Matrix (SPDM) when computing ${\phi }_{c}(G)$. The formula in Eq. (10) is conceptualized based on the motivation of evaluating error performance in graph networks. It is defined as the ratio of the difference between the true value and the calculated value to the true value (or original value).

Closeness Centrality and Dangling Centrality both utilize the Shortest-Path Distance Matrix (SPDM) to assess a node’s importance in a network, but they differ in the steps and interpretation of their calculations. In **Closeness Centrality**, the process starts by computing the SPDM, followed by summing the elements of the matrix. Then, the element-wise inverse of the SPDM is taken, providing a measure of centrality based on the inverse of the distances to all other nodes. On the other hand, **Dangling Centrality** also begins with the computation of the SPDM, but its second step involves taking the element-wise inverse of SPDM excluding the 0 entries (those that represent self-loops or non-existent paths). Finally, the sum of the element-wise inverse values is computed. The key difference lies in Dangling Centrality’s focus on measuring the impact of a node’s absence: it first computes the Dangling Centrality for the original network, then repeats the process after removing the node’s edges, yielding a new SPDM. The Dangling Centrality for a node *i* is then calculated as the difference between its centrality in the original and the modified network, normalized by the original centrality (see Eq. 10). Thus, Dangling Centrality captures the relative importance of a node by assessing the disruption caused by its absence, unlike Closeness Centrality, which purely evaluates the proximity of nodes in terms of network distances.

9 $${\phi }_{c}(G)=\sum_{G}\sum_{i\ne j\in G}{\left(\frac{1}{SPDM}\right)}_{neglecting 0 values}=\sum_{G}\sum_{i\ne j}{(SPDM)}_{element-wise}^{-1}$$

Neglecting the 0 value of shortest-path refers to ignoring self-connections or initially disconnected nodes in the shortest path calculations. In a network graph, self-connections (where a node has a path to itself) and nodes that are initially disconnected do not contribute to strong network communication. Therefore, for Dangling Centrality, the 0 values in the shortest path matrix are ignored, as they do not reflect the flow or disruption of information within the network. The measure Dangling Centrality $\left({\phi }_{c}\right)$ for a single node $({\varvec{i}})$ was computed using Eq. (10).

10 $${\phi }_{c}\left(i\right)=\frac{{\phi }_{c}\left(G\right)-{{\varvec{\phi}}}_{{\varvec{c}}}({{\varvec{G}}}_{{\varvec{i}}})}{{\phi }_{c}\left(G\right)}$$

In Eq. (10) the term ${{\varvec{\phi}}}_{{\varvec{c}}}({{\varvec{G}}}_{{\varvec{i}}})$ is a key parameter in computation of Dangling Centrality ${{\varvec{\phi}}}_{{\varvec{c}}}\left({{\varvec{G}}}_{{\varvec{i}}}\right)$ for a single node by making corresponding node $({\varvec{i}})$ degree centrality **(DC)** equals to zero. The example of five vertices undirected graph Fig. 1a is considered. For computation of parameter ${{\varvec{\phi}}}_{{\varvec{c}}}({\varvec{G}})$, **Shortest-Path Distance Matrix (SPDM)** was considered and calculated using Eq. (3).

11 $$\phi_{c} \left( G \right) = \mathop \sum \limits_{G} \mathop \sum \limits_{i \ne j \in G} { }\left[ {\begin{array}{*{20}c} \infty & {1.00} & {\begin{array}{*{20}c} {1.00} & {1.00} & {0.5} \\ \end{array} 00} \\ {1.00} & \infty & {\begin{array}{*{20}c} {1.00} & {0.500} & {0.334} \\ \end{array} } \\ {\begin{array}{*{20}c} {1.00} \\ {1.00} \\ {0.500} \\ \end{array} } & {\begin{array}{*{20}c} {1.00} \\ {0.500} \\ {0.334} \\ \end{array} } & {\begin{array}{*{20}c} {\begin{array}{*{20}c} \infty \\ {0.500} \\ {0.334} \\ \end{array} } & {\begin{array}{*{20}c} {0.500} \\ \infty \\ {1.00} \\ \end{array} } & {\begin{array}{*{20}c} {0.334} \\ {1.00} \\ \infty \\ \end{array} } \\ \end{array} } \\ \end{array} } \right] = { 14}.{336}$$

12 $${\phi }_{c}\left(G\right)=7.167$$

In Eq. (12), the overall summation of the element-wise inverse of the Shortest Path Distance Matrix (SPDM) is normalized by dividing by 2. This normalization step ensures that the resulting Dangling Centrality values are appropriately adjusted for undirected graphs, where each connection is counted twice, once for each direction. By dividing by 2, the measure reflects the true importance of the node in the network, maintaining consistency with standard practices in centrality calculations for undirected graphs.

After the calculation of SPDM, **“Dangling Centrality **$\left({{\varvec{\phi}}}_{{\varvec{c}}}\right)$**”** was calculated for **Node ID 0** (presented in Table 6). After turning ${\varvec{D}}{\varvec{C}}=0$ for Node ID 0, the network configuration is disturbed and there was no communication between many nodes in a graph as mentioned in Fig. 1a, b. Therefore, ${\phi }_{c}({G}_{0})$ is computed using Eq. (13) by turning row 1 and column 1 (in SPDM matrix) equals to zero. Moreover, the remaining nodes (in the same SPDM matrix) demonstrate no links between other Nodes IDs passing through Node ID **0**.

13 $${\phi }_{c}\left({G}_{0}\right)=\sum_{G}\sum_{i\ne j\in G}{\frac{1}{ \left[\begin{array}{ccc}0& 0& \begin{array}{ccc}0& 0& 0\end{array}\\ 0& 0& \begin{array}{ccc}1& 0& 0\end{array}\\ \begin{array}{c}0\\ 0\\ 0\end{array}& \begin{array}{c}1\\ 0\\ 0\end{array}& \begin{array}{ccc}\begin{array}{c}0\\ 0\\ 0\end{array}& \begin{array}{c}0\\ 0\\ 1\end{array}& \begin{array}{c}0\\ 1\\ 0\end{array}\end{array}\end{array}\right]}}_{neglecting 0 in shortest-walk length}$$

As a result, the following changes were observed;

- Matrix in denominator of Eq. (13) clears the loss of Node ID 0 that effects the communication between various nodes of network.
- $i\ne j$ belongs to graph (G) indicates to avoid the diagonal elements of SPDM.
- 0 element present in SPDM indicates there is no link or exchange between two nodes ids that are ${\varvec{i}} and {\varvec{j}}.$ Therefore after calculating inverse of each element present in SPDM 0 walk lengths are avoided.
  14 $${\phi }_{c}\left({G}_{0}\right)=2$$

The Dangling Centrality for Node ID **0** will be computed as shown in Eqs. (15) and (16):

15 $${\phi }_{c}\left(0\right)=\frac{7.167-2}{7.167}$$

16 $${\phi }_{c}\left(0\right)=0.7209$$

This process is visually represented in Fig. 1a, b, where the network is plotted both before and after the removal of the node’s connections. Additionally, Eq. (13) provides the computation for Node ID 0, showing how the removal of its connections alters the degree of other nodes that remain in the network. This approach reflects the disruption caused by the absence of the node links and helps in evaluating its centrality within the network.

Table 7 presents a comprehensive comparison of the six centrality metrics. The analysis reveals the level of influence associated with specific Node IDs within the graphs depicted in Fig. 1a, b. It highlights the identification of nodes that play a more pivotal role and examines the dependencies of other nodes on these influential ones for effective communication within the network. For instance, Node ID 0 for Fig. 1a shows the highest centrality values for all measures and Node ID 2 for an undirected graph in Fig. 1b shows the highest centrality values for all measures.

**Table 7:** Comparative chart examining centrality metrics in two unique small graphs.

Node IDs		Centrality Metrics											
Figure 1a	Figure 1b	DC		CC		BC		EVC		KC		${{\varvec{\phi}}}_{{\varvec{c}}}$	
**0**	**1**	3	2	0.2	0.1250	8	0	0.6071	0.2726	2.0388	1.0208	0.72093	0.2800
**1**	**2**	2	4	0.1428	0.1667	0	3.6667	0.4995	0.4728	1.7597	1.0413	0.3953	0.3733
**2**	**3**	2	3	0.1428	0.1428	0	0.6667	0.4995	0.3941	1.7597	1.0311	0.3953	0.3200
**3**	**4**	2	4	0.1667	0.1667	6	1.3333	0.3401	0.5000	1.6748	1.0414	0.58139	0.3600
**4**	**5**	1	4	0.111	0.1667	0	3.6667	0.1573	0.4728	1.3350	1.0413	0.30232	0.3733
**–**	**6**	–	3	–	0.1428	–	0.6667	–	0.3942	–	1.0311	–	0.3200

**Fig. 3:**
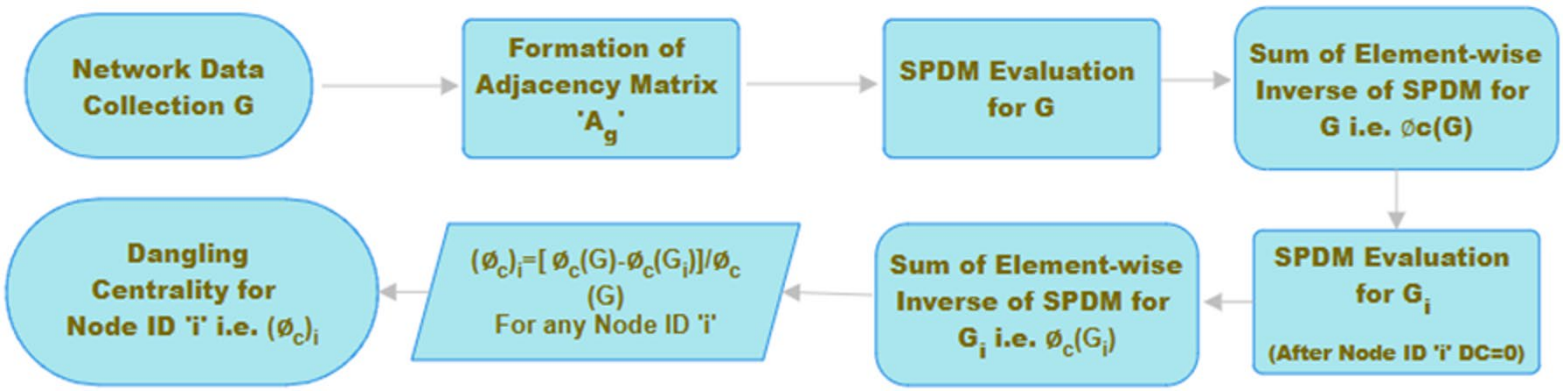
Flowchart of dangling centrality evaluation.

**Fig. 4:**
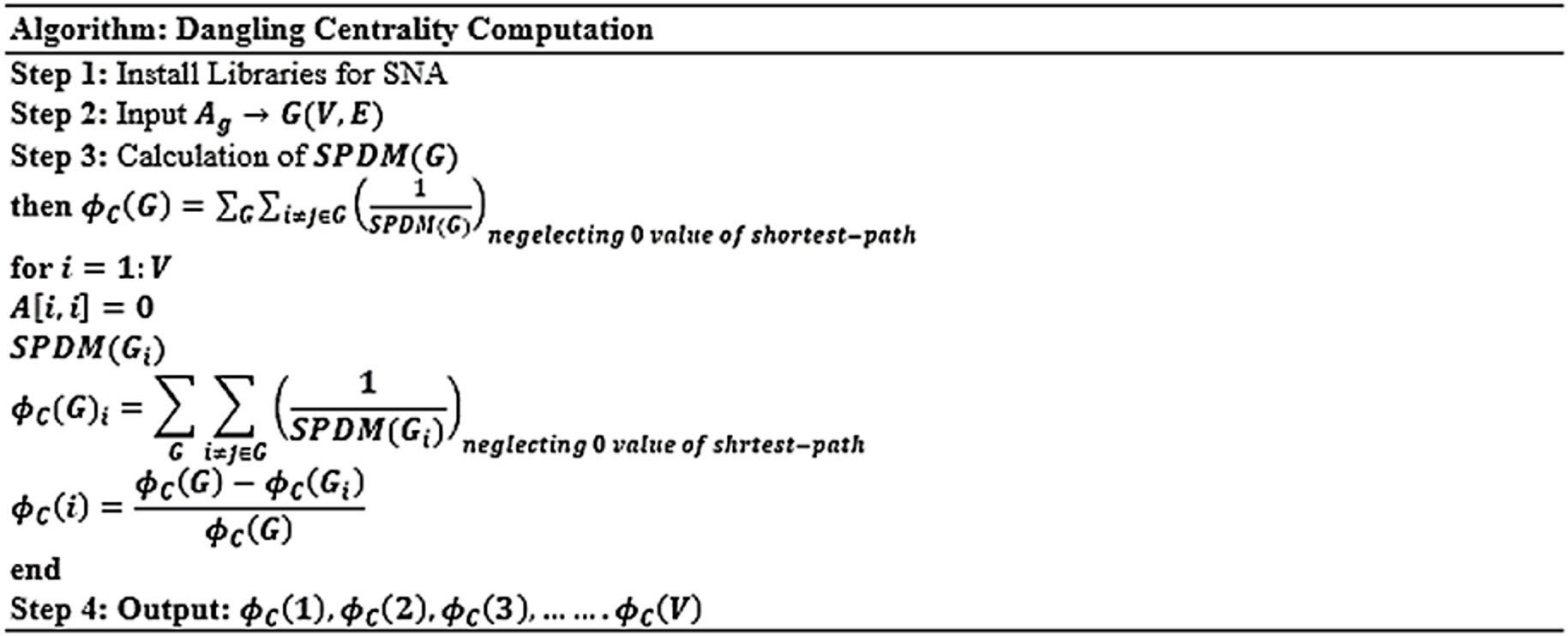
Pseudo-code for novel dangling centrality metric.

Table 7 presents the calculated centralities metric for two minor graphs, including the Dangling Centrality metric. Furthermore, Table 8 displays Pearson’s, Spearman’s, and Kendall’s correlation coefficient values, indicating a robust positive correlation between the dangling centrality and all five traditional centrality metrics: DC, BC, CC, EVC, and KC. This demonstrates that the newly introduced Dangling centrality metrics exhibit a robust association and will play a crucial role in identifying essential individuals, nodes, or products within any large and intricate real-life network graph. Figures 3 and 4 depicts the flowchart and introduced algorithm for **Dangling Centrality **$\left({{\varvec{\phi}}}_{{\varvec{c}}}\right)$ metric.

**Table 8:** Pearson’s, Spearman’s, and Kendall’s correlation coefficient between dangling centrality and 5 popular centrality metrics.

	Small Dataset	Centrality Metrics	DC	BC	CC	EVC	KC
Pearson’s Correlation Coefficient	5 nodes graph	Dangling Centrality ${{\prime}\varnothing }_{c}{\prime}$	0.8758113	0.9671900	0.9803829	0.5968716	0.8754605
	6 nodes graph		0.9897783	0.8882636	0.9911944	0.9566603	0.9897083
Spearman’s Correlation Coefficient	5 nodes graph		0.9176629	0.9176629	1.0000000	0.6842105	0.6842105
	6 nodes graph		0.9534626	1.0000000	0.9534626	0.8060599	0.8181818
Kendall’s Correlation Coefficient	5 nodes graph		0.8819171	0.8819171	1.0000000	0.5555556	0.5555556
	6 nodes graph		0.9198662	1.0000000	0.9198662	0.6671244	0.6923077

Table 6 clears that **Node ID 0** is the most influential node in a graph for communication and maintaining connection between different parts of graph as it also have maximum “Dangling centrality” like other centrality measures shown in Table 7.

In the next section, we delved into the examination of a novel centrality measure called **Dangling centrality** $\left({{\varvec{\phi}}}_{{\varvec{c}}}\right)$, utilizing a real-life datasets. The analysis will focus on understanding how the removal of a vertex, node, person, protein, or customer can impact network communication dynamics. This exploration aims to shed light on the crucial role of individual components in constructing a robust network graph of information.

## Exploring the use of centrality measures in large-scale network analysis

**Fig. 5:**
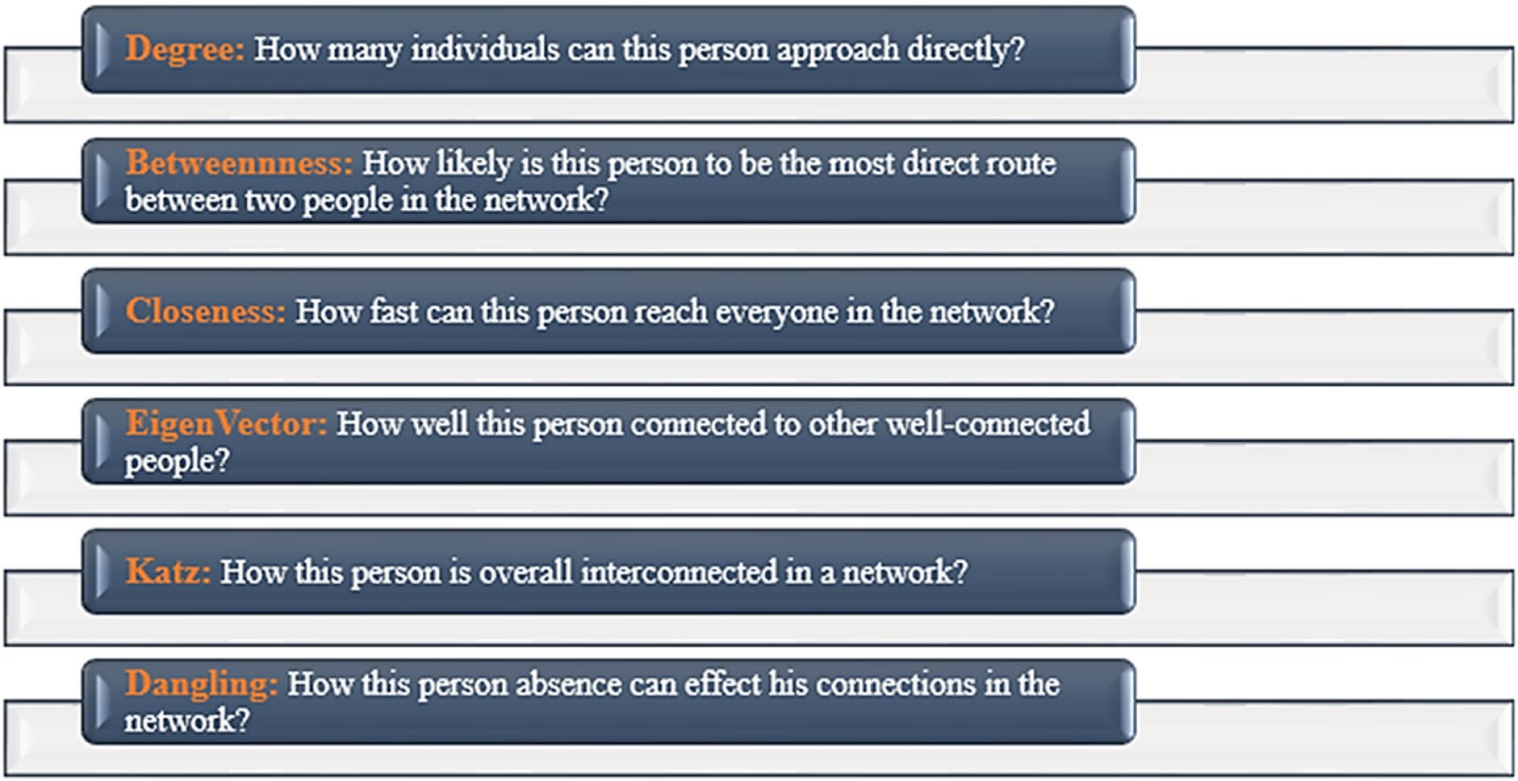
Centrality metrics phenomena in Social Network Analysis (SNA).

Centrality metrics stand out as frequently employed instruments in real-world scenarios for deriving insights from extensive network datasets. A concise discussion on these measures and their practical applications is presented in Fig. 5.

There are two large product network datasets analyzed through different Centrality metrics (CM) for determination of influential products in marketing and griping the interest of customers that are discussed in the following section.

### Amazon datasets (Amazon product co-purchasing network)

1. The first dataset composed of or $(262111\boldsymbol{ }{\varvec{n}}{\varvec{o}}{\varvec{d}}{\varvec{e}}{\varvec{s}},1234877\boldsymbol{ }{\varvec{e}}{\varvec{d}}{\varvec{g}}{\varvec{e}}{\varvec{s}})$**,** was collected accessing Amazon webpages on 2nd March 2003 (see Table 9). It deals with “Clients Who Purchase This Product Also Purchase” article of the Amazon webpage. If an item ‘***i’*** is repeatedly co-purchased with item ‘***j’***, which is in directed graph demonstrated by edge ‘***i’*** to ***‘j’***^42^.

**Table 9:** Dataset Statistics of Amazon Website March 2003.

Nodes	262,111
Edges	1,234,877
Description	Amazon product co-purchasing network from March 2 2003
Source	^42,43^
Link	https://snap.stanford.edu/data/amazon0302.html
2. Second data comprised of $(403394\boldsymbol{ }{\varvec{n}}{\varvec{o}}{\varvec{d}}{\varvec{e}}{\varvec{s}},3387388\boldsymbol{ }{\varvec{e}}{\varvec{d}}{\varvec{g}}{\varvec{e}}{\varvec{s}})$ was considered for assessment of “Amazon product dataset” that dated June 2003 (see Table 10). This data shared the information regarding behavior of frequent buyer in terms of purchasing products in combination^43^. Dataset was analyzed through organized approaches. First approach was transformation in ***“Adjacency Matrix (Ad)”*** and the second approach was “***formation of graph”.***

**Table 10:** Dataset Statistics of Amazon Website June 2003.

Nodes	403,394
Edges	3,387,388
Description	Amazon product co-purchasing network from June 1 2003
Source	^42,43^
Link	https://snap.stanford.edu/data/amazon0601.html

### Bitcoin dataset

This cryptocurrency dataset $(\text{5,881}\mathbf{n}\mathbf{o}\mathbf{d}\mathbf{e}\mathbf{s},\text{35,592}\mathbf{e}\mathbf{d}\mathbf{g}\mathbf{e}\mathbf{s})$ represents a connected graph of individuals engaged in Bitcoin transactions on a platform known as "Bitcoin over-the-counter (OTC)," resembling a network where users express trust or skepticism towards one another. Due to the anonymity of Bitcoin users, maintaining a record of users’ reputations is crucial to prevent transactions with potentially fraudulent or unsafe individuals. Participants in the Bitcoin OTC platform assign trust levels to others on a scale ranging from − 10 (complete distrust) to + 10 (complete trust), with increments of 1 (see Table 11). This network dataset serves as the primary explicitly weighted and labeled graph available for research purposes^42,44^.

**Table 11:** Dataset Statistics of Bitcoin OTC web of trust network.

Nodes	5881
Edges	35,592
Description	Bitcoin OTC web of trust network
Source	^44^
Link	https://snap.stanford.edu/data/soc-sign-bitcoin-otc.html

### Yeast protein–protein interaction graph: dataset

An interaction dataset $(2361\boldsymbol{ }{\varvec{n}}{\varvec{o}}{\varvec{d}}{\varvec{e}}{\varvec{s}},7182\boldsymbol{ }{\varvec{e}}{\varvec{d}}{\varvec{g}}{\varvec{e}}{\varvec{s}})$ of Saccharomyces Cerevisiae (budding yeast) proteins is employed for centrality measure computation to identify key proteins. The network comprises 2361 nodes, representing yeast proteins, connected by 7182 directed and unweighted edges that indicate physical interactions (see Table 12). Additionally, there are 536 loops within the network^31^.

**Table 12:** Dataset statistics of PPI network.

Nodes	2361
Edges	7182
Description	Protein–Protein Interaction (PPI) Network
Source	^8,31^
Link	https://api.semanticscholar.org/CorpusID:219684564

### Unveiling interactions: adjacency matrix analysis of PPI network dataset

**Fig. 6:**
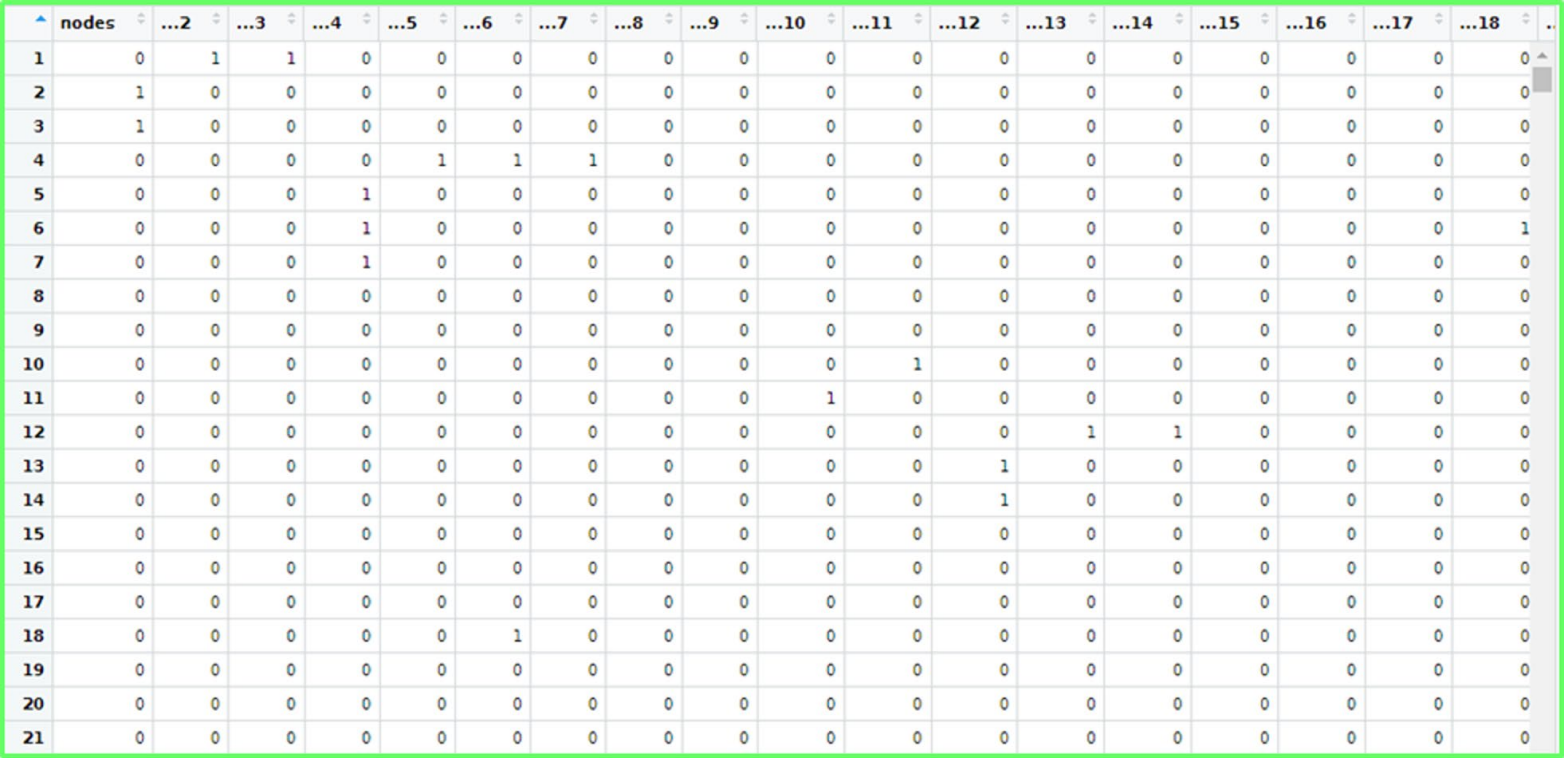
Sample of Adjacency matrix formation for PPI network dataset in R.

As mentioned earlier, first approach was that “Product network dataset” of Amazon website is converted into “***Adjacency Matrix ***${(\boldsymbol{ }{\varvec{A}}}_{{\varvec{g}}})$***”*** for the analysis of dataset, shown in Fig. 6. The matrix ${A}_{g}$ is taken as input for computation of centrality metrics and other measures of network analysis for catching significant proteins (node) in considered PPI network.

**Fig. 7:**
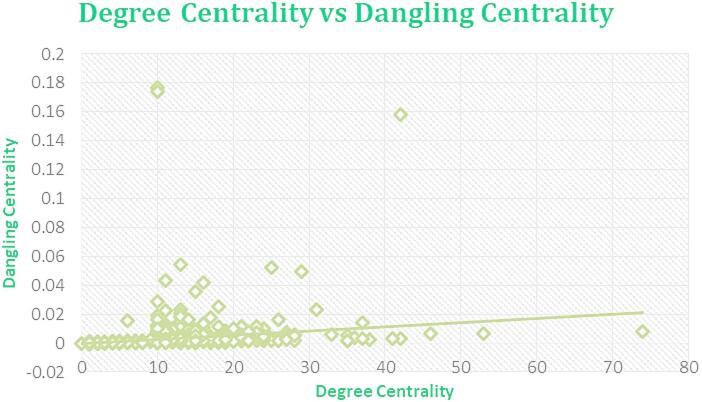
Regression plot between Dangling Centrality and DC for Amazon Network June 2003.

**Fig. 8:**
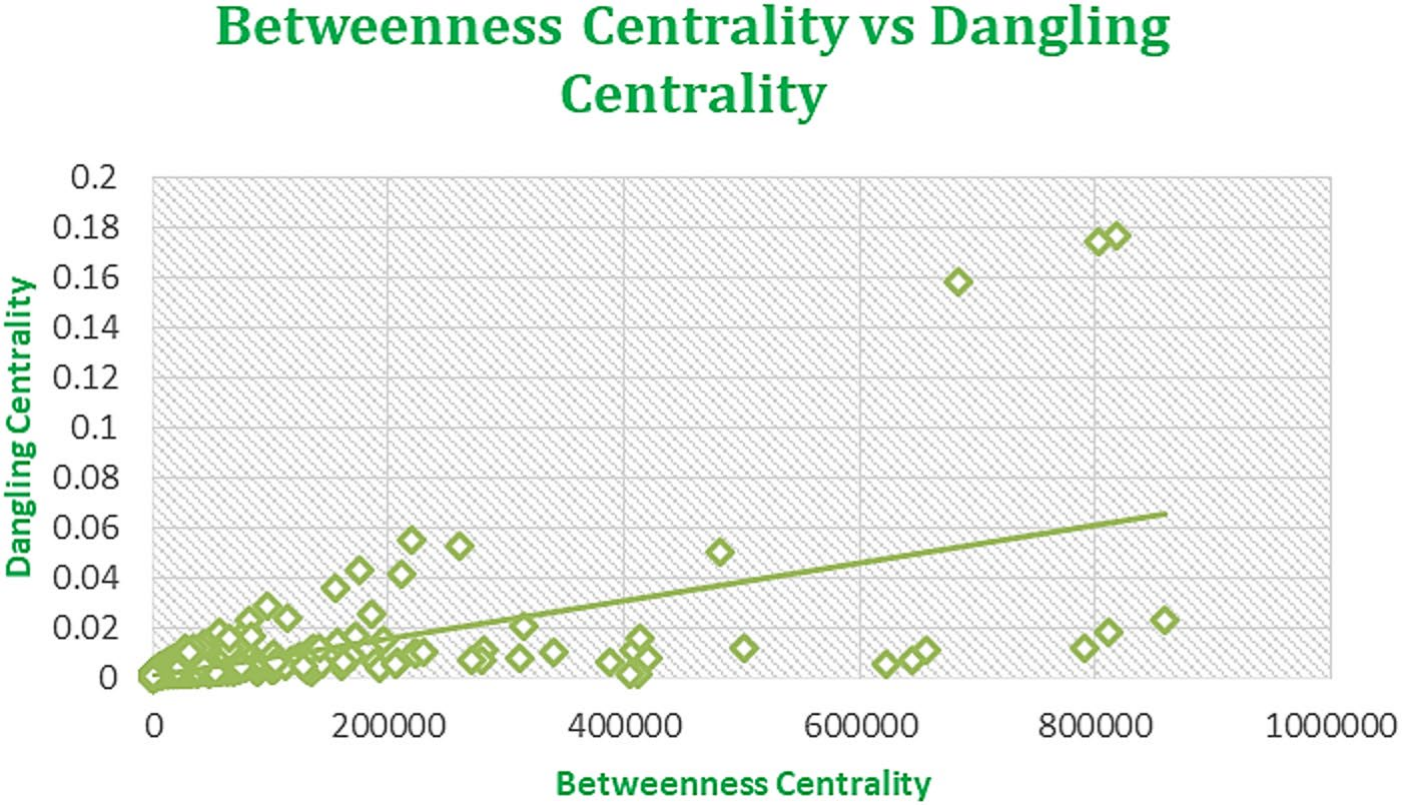
Regression plot between Dangling Centrality and BC for Amazon Network June 2003.

One of the basic tool of SNA, Centrality measures (CM) was discussed comprehensively in this section and this method was replicated on two small datasets. Next section contains discussion on more tools of Social Network Analysis (SNA) for large real-life Datasets and its comparison with novel Dangling Centrality metric.

### Centrality in context: understanding various real-life networks through graph formation and analysis: results and discussion on comparison analysis of our SOTA ${{^{\prime}}{\varnothing }}_{{\varvec{c}}}$’

**Fig. 9:**
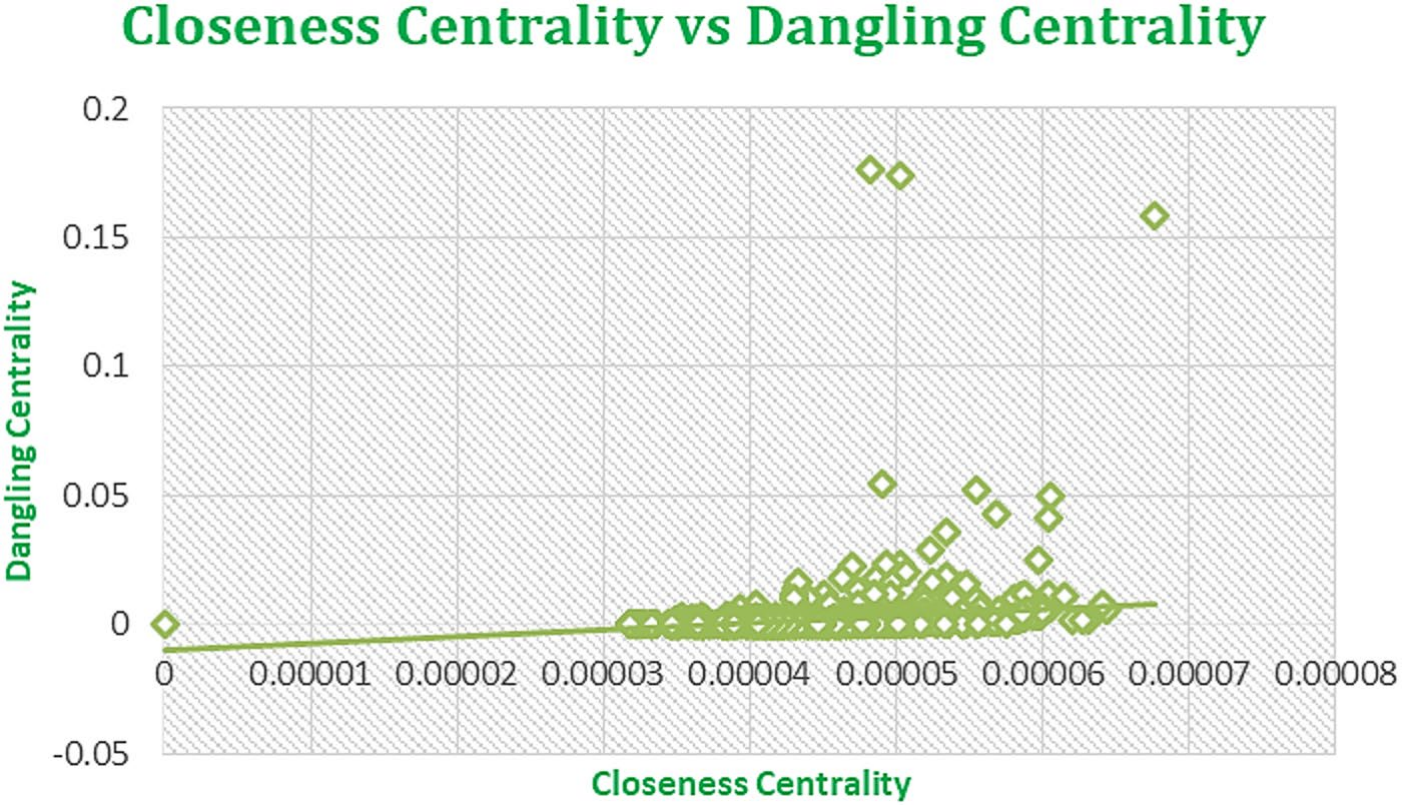
Regression plot between Dangling Centrality and CC for Amazon Network June 2003.

PPI network data of 2361 proteins (nodes) can be seen in Fig. 9 and for understanding the influence of proteins in the considered network dataset, centrality metrics are measured for 2361 proteins (nodes) of PPI network, outcomes are demonstrated in Tables 13 and 14 for 2 Amazon product networks, one PPI network and Bitcoin crypto currency network datasets.

**Fig. 10:**
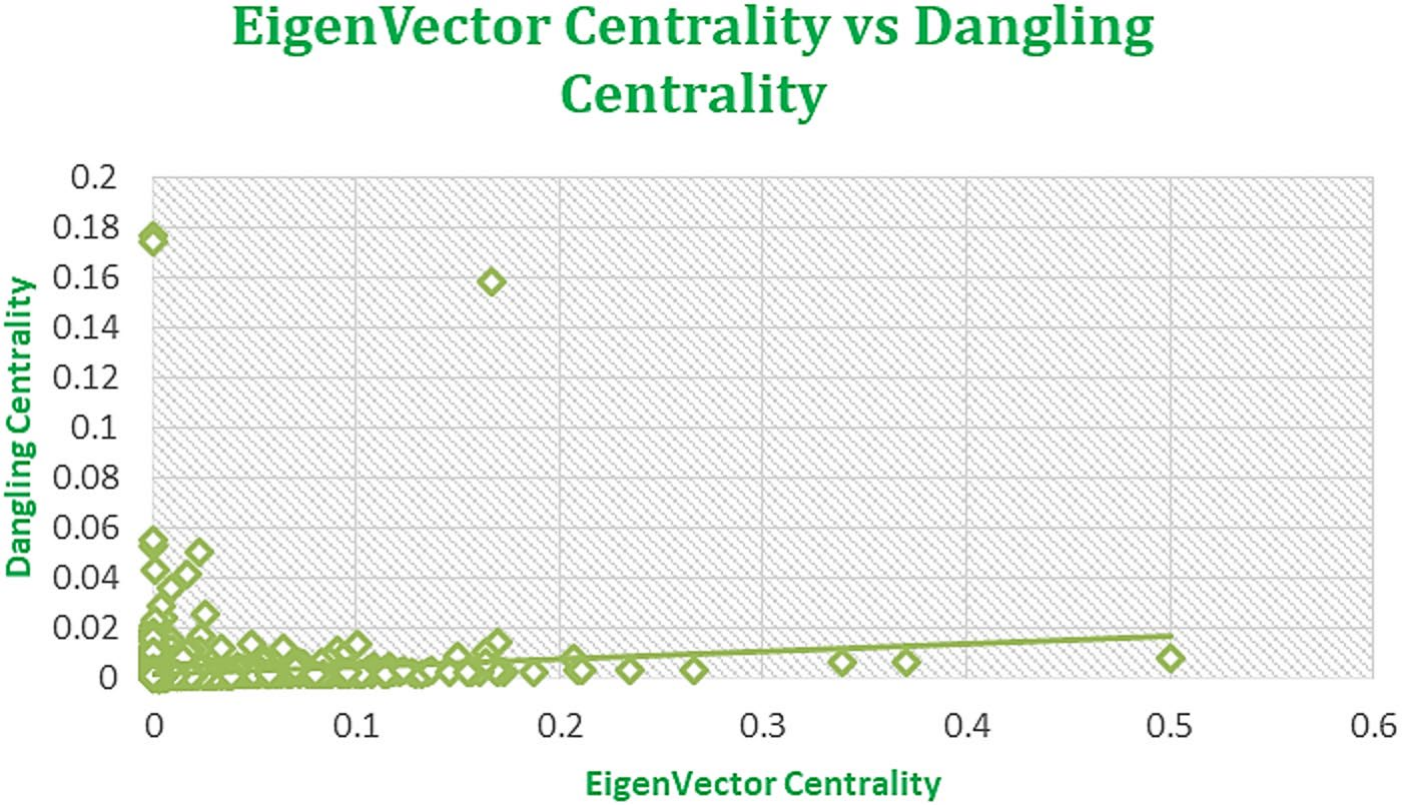
Regression plot between Dangling Centrality and EVC for Amazon Network June 2003.

**Fig. 11:**
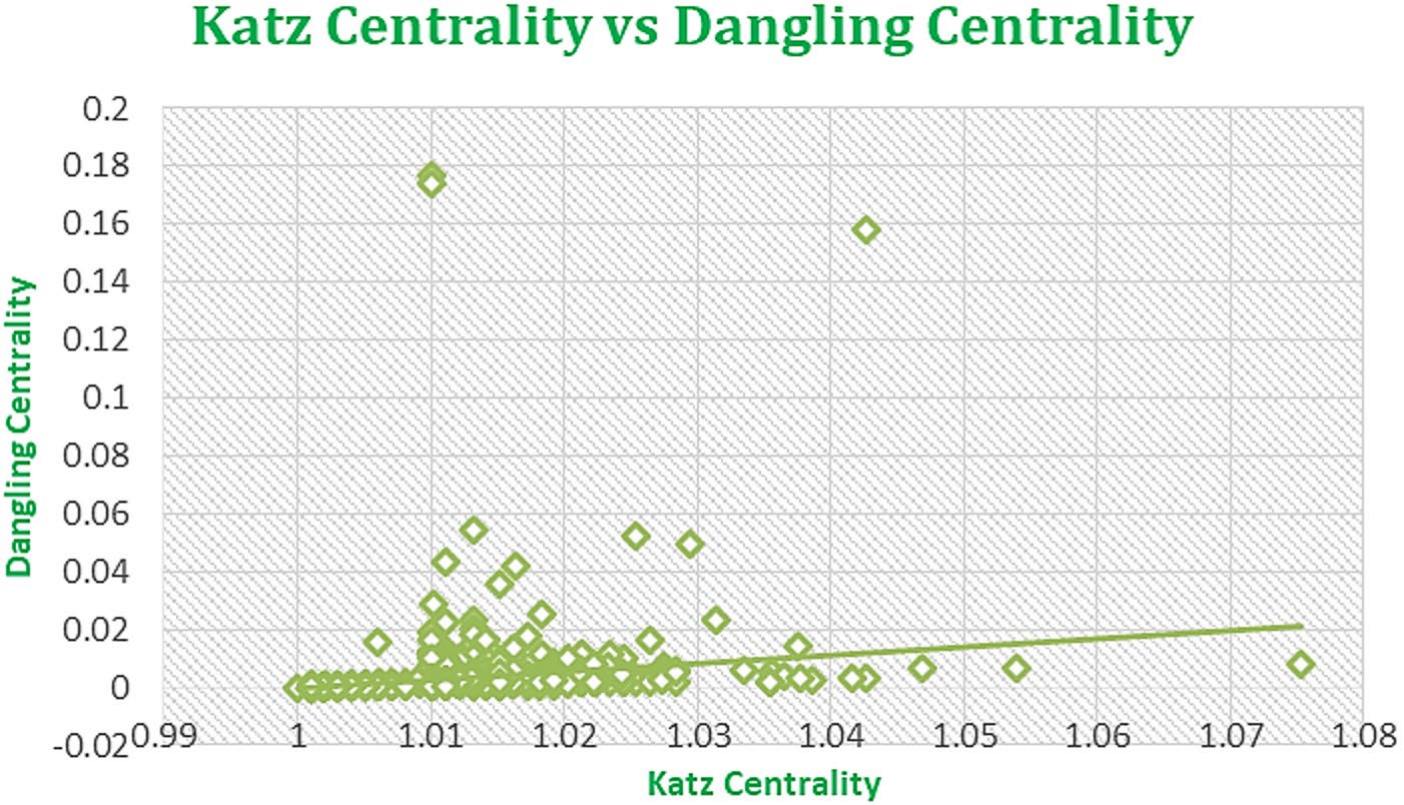
Regression plot between Dangling Centrality and KC for Amazon Network June 2003.

**Table 13:** Six centrality metrics for the Amazon complex product network.

Amazon Product Network March 2003						
Products (Nodes Id)	Degree (DC)	Betweenness $(BC \times {10}^{-3})$	Closeness $(CC \times {10}^{5})$	Eigenvector ($EVC\times {10}^{4})$	Katz (KC)	Dangling ${\varnothing }_{c}({10}^{3})$
0	5	1.2676	9.4984	291.6300	1.0550	2.1400
9	44	285.2227	11.7952	5000.0000	1.4912	45.0319
499	5	2.6992	7.0811	14.6399	1.0529	0.09724
500	6	5.9161	8.2610	16.9986	1.0650	3.1389
1000	4	0	6.3383	6.7448	1.0409	0.4031

**Table 14:** Computation of six centrality metrics using Large Amazon product graph.

Amazon Product Network June 2003						
Products (Nodes Id)	Degree (DC)	Betweenness $(BC \times {10}^{-3})$	Closeness $({\varvec{C}}{\varvec{C}} \times {10}^{5})$	Eigenvector (${\varvec{E}}{\varvec{V}}{\varvec{C}})$	Katz (KC)	Dangling ${\varnothing }_{{\varvec{c}}}({10}^{3})$
0	10	0.4524	5.1939	0.0863	1.1244	1.0877
5	74	311.7653	6.0335	0.5000	1.8879	7.8763
29	31	858.7171	5.0241	0.0012	1.3649	23.1655
500	10	3.8162	4.7700	0.0007	1.1140	1.4639
1000	10	0	0.0124	1.0142e-17	1.0000	0

Analysis was conducted on the dataset comprising 1001 products from the Amazon website, employing six primary centrality metrics to identify the most crucial nodes. The results, as illustrated in Table 13, revealed strong interconnections among all six measures. Notably, Node ID 9 emerged as significant across all metrics colored with pink. For the extensive dataset of another Amazon website, encompassing “1001 Nodes,” computations for all six centrality metrics were performed, as evident in Table 14. These calculations aimed to identify pivotal nodes (products) within the graph representing the "Amazon website." Results directed that **Node ID 5 (highlighted in yellow color)** in Amazon product network was an important product because it has larger figure of above six centrality metrics, which showed that this node was an essential in concept of business strategies. Additionally, **Node ID 29 (highlighted in green color)** was ranked as the second highest in centrality metric calculation, as it also played an important role in business that carried out on Amazon product website graph.

From Table 15 outcomes protein/Node ID 1443 that is **YKU80 (YMR106C)** indicates that this will play a crucial role due to highest centrality metrics outcomes, also this is observed through literature by actively participating in the recovery and repair of enzymes with restricted functionality and the DNA double-strand break pathway. Its primary function involves safeguarding these pathways from the potential introduction of errors, thus contributing to the maintenance of genomic integrity. Notably, this yeast protein stands out with significant importance, as evidenced by its highest centrality metrics among a dataset of 2361 proteins engaged in protein–protein interactions^31,45^.

**Table 15:** Computation of six centrality metrics for a graph comprising 2361 yeast proteins.

Protein–Protein interaction network						
Proteins (Nodes Id)	Degree (DC)	Betweenness $$(BC \times {10}^{-4})$$	Closeness $$(CC \times {10}^{6})$$	Eigenvector ($$EVC)$$	Katz (KC)	Dangling $${\varnothing }_{c}({10}^{3})$$
1	2	0	0	0.0005	1.0020	0.7709
147	57	21.0631	3.0457	0.1523	1.6579	11.4389
209	62	19.3335	3.0473	0.0563	1.7009	11.9993
302	64	17.5064	3.0479	0.4396	1.7740	7.0325
492	56	11.9037	3.0475	0.4368	1.6883	6.4669
566	64	19.7100	3.0475	0.0719	1.7225	10.1602
784	62	20.6946	3.0480	0.1275	1.7041	10.9563
1443	63	20.7643	3.0476	0.0641	1.7047	13.7943
2361	1	0	3.0118	0.0005	1.0010	0.7709

**SEC27 Node ID 209 in the considered PPI dataset**, a component of the Coatomer Complex (YGL137W), holds significant importance in the literature due to its integral role in various cellular processes. Notably, it shares a substantial 45% sequence resemblance with the mammalian coatomer subunit beta. Functionally, SEC27 is responsible for encoding membrane proteins essential for Golgi transportation, acting in conjunction with ARF1 for endoplasmic reticulum (ER) processes. Moreover, it actively participates in the initial steps of protein sorting in yeast, particularly for endosomal proteins. Its prominence is underscored by the fact that, within a dataset of 2361 proteins engaged in protein–protein interactions, SEC27 ranks almost second highest in centrality metrics^45,46^.

Continuing the exploration of yeast protein significance, the third highest protein in centrality metrics within the dataset of 2361 proteins is Srp1 i.e. Node ID 147 that is clear from Table 15. This protein introduces a novel approach to protein degradation and serves as a distinctive signal receptor in the context of nuclear localization. The absence of Srp1 has profound consequence leading to cellular mortality, emphasizing its indispensable role in maintaining vital cellular functions and processes^45^.

Table 16 serves as a computation reference for six centrality metrics applied to the intricate Bitcoin dataset. Notably, it is evident that both Node ID 1 and Node ID 2 exhibit the highest measures across all metrics. Additionally, the outcomes of the novel metric align with the results obtained from the established centrality metrics. Node ID 1 and Node ID 2 emerge as the focal points of highest centrality metrics within the Bitcoin network, symbolizing the most connected or popular entities in the realm of cryptocurrency transactions as seen in Table 16 results. These nodes serve as key hubs, indicative of their prominence and extensive connections in the intricate web of cryptocurrency dealings.

**Table 16:** Centrality computations for Bitcoin large dataset.

Products (Nodes Id)	Degree (DC)	Betweenness $$(BC \times {10}^{-4})$$	Closeness $$(CC \times {10}^{6})$$	Eigenvector ($$EVC)$$	Katz (KC)	Dangling $${\varnothing }_{c}({10}^{3})$$
1	750	48.8032	1.7728	0.1508	1.7883	67.1201
2	754	27.7906	1.7738	0.5000	1.8544	13.5501
500	25	0.2223	1.7625	0.0182	1.0317	1.3792
1000	4	0	1.7608	0.0015	1.0044	0.2918

Centrality measures, including the proposed dangling centrality, provide valuable insights into network dynamics by identifying critical nodes and their roles. Traditional metrics highlight nodes based on connectivity or influence, while dangling centrality uniquely evaluates the network’s resilience to communication loss. This allows for the preemptive design of robust systems by analyzing the impact of a node’s link elimination. For instance, if communication with a key node is disrupted, alternative pathways can be strategically designed to maintain the network’s functionality, ensuring minimal disruption in complex systems like Bitcoin or other interconnected networks.

The proposed dangling centrality measure is strongly related to almost all the centrality metrics in the literature which is seen in Table 17. Dangling centrality plays a vital role in the study of large networks by knowing the essentiality of nodes. Results on different examples like simple networks in Fig. 2a, b, amazon website datasets of different months and some other large data are considered like bit-coin data and disease spread data is also extracted from SNAP (**Stanford Large Network Dataset**^1^** Collection)** and inferred. This work mainly focus on product networks like amazon website to promote “Business Intelligence” and biological dataset to discuss essentiality of centrality metrics in various real life domains.

**Table 17:** Novel Dangling Centrality comparison with popular centralities through Pearson’s, Spearman’s and Kendall’s Correlation Coefficient.

	Real-life dataset	Centrality metrics	DC	BC	CC	EVC	KC
Pearson’s Correlation Coefficient	Amazon 1	Dangling Centrality ${{\prime}\phi }_{c}{\prime}$	0.4620371	0.8154608	0.4049604	0.3679921	0.4650667
	Amazon 2		0.2878921	0.6481128	0.2333661	0.1278617	0.2872377
	PPI		0.7812787	0.8922564	0.2676383	0.3739042	0.7794644
	Bitcoin		0.7855071	0.8896052	0.06693472	0.5780862	0.7710929
Spearman’s Correlation Coefficient	Amazon 1		0.7554034	0.8982081	0.6932152	0.6855223	0.7470634
	Amazon 2		0.7787221	0.8381494	0.6618990	0.5943911	0.7933662
	PPI		0.6999998	0.7105681	0.5552777	0.6450815	0.7387394
	Bitcoin		0.6919743	0.8620611	0.5272577	0.5709354	0.6787683
Kendall’s Correlation Coefficient	Amazon 1		0.5630540	0.7392368	0.4982697	0.4933784	0.5286709
	Amazon 2		0.6030667	0.6894940	0.4818794	0.4373180	0.5750534
	PPI		0.6008932	0.5924809	0.4555893	0.4772644	0.5659789
	Bitcoin		0.5945892	0.8217732	0.4249878	0.4574984	0.5533833

The correlation with existing centrality measures is conducted to show that Dangling Centrality does not completely diverge from traditional metrics, confirming its relevance in centrality analysis. However, its computation and decision-making process are different. Unlike traditional centrality measures, Dangling Centrality evaluates the impact of removing all links of a node (i.e., reducing its degree to zero) rather than the node’s outright removal. This allows Dangling Centrality to capture how the loss of communication from a node affects the overall network, providing insights that other measures may miss. By focusing on the disruption caused by a node’s loss of connectivity, Dangling Centrality identifies nodes that play a critical role in the network’s communication flow, which is particularly useful for understanding network robustness and identifying potentially vulnerable points.

Figures 7, 8, 9, and 10 displaying regression plots on R, each depicting the positive and strong associations between our **SOTA Dangling Centrality and well-established centrality measures**, including DC, BC, CC, EVC, and KC. These plots represent the complex dataset of Amazon Product Network of June 2003, showcasing the positive and strong associations between Dangling Centrality and established centrality measures such as DC, BC, CC, EVC, and KC. The analyses emphasize the significant positive correlation observed with the novel centrality measure in the intricate context of Amazon data^1^.

The proposed methodology demonstrates a strong positive correlation, as shown in Figs. 7, 8, 9, 10 and 11, indicating that Dangling Centrality aligns with established centrality measures like DC, BC, CC, EVC, and KC. This suggests that Dangling Centrality is consistent with traditional methods while introducing a critical new perspective. Unlike existing metrics that evaluate the importance of a node based on its presence, Dangling Centrality assesses its significance by considering the impact of its communication absence. This novel approach is crucial for policymakers, as neglecting such nodes or factors can disrupt communication across the entire network, making it essential for effective system design and planning.

The work on Dangling Centrality differs from Dynamic Age in that, it focuses on the disruption of network communication and information flow when a node’s links are removed, rather than just assessing the change in the largest eigenvalue of the adjacency matrix^47^. The comparison between Dynamic Age and Dangling Centrality has been updated. Both measures evaluate the node’s importance by assessing the disruption caused when a node is removed from the network.

## Limitations of the proposed method

Dangling Centrality can be studied first to assess the absence or loss of key entity links in a network. By identifying alternative nodes and their roles, this metric helps in decision-making to avoid communication failures in the system, offering a more proactive approach compared to traditional metrics. However, it has some limitations:

Increased Computational Time for Large Networks:

As the network size increases, the computational time for calculating Dangling Centrality slightly increases, which may affect its efficiency for very large-scale networks.

Domain Expertise Required for Decision Making:

To effectively utilize Dangling Centrality across diverse domain datasets, specialized knowledge is required to identify which entity edges/links, when removed, would disrupt network communication and stability.

Complexity in Assessing Communication Disruption:

The metric may not easily identify which node communication absences will lead to significant communication breakdowns, as this depends on the specific context and structure of the network, requiring deep domain understanding for accurate assessment.

## Conclusions

This research work introduced the novel **Dangling Centrality** $({{\varvec{\phi}}}_{{\varvec{C}}})$ metric and evaluated its effectiveness using two “Amazon product networks,” a PPI network, and a Bitcoin network dataset. The input data was mined and transformed into adjacency matrices for analyzing social network structures. Our analysis revealed a robust connection between Dangling Centrality and five established centrality metrics—DC, BC, CC, EVC, and KC. However, while Pearson’s, Spearman’s, and Kendall’s correlation coefficients were applied to confirm that Dangling Centrality results align with existing literature metrics, the conceptual utilization $({{\varvec{\phi}}}_{{\varvec{C}}})$ of diverges significantly. Unlike traditional metrics, Dangling Centrality focuses on the impact of the absence of node links, products, proteins, or individuals in disrupting network communication. This distinct approach was demonstrated through the study of four real-world datasets and two simple network graph examples. The results highlight the complementary role of Dangling Centrality, particularly in pre-designed networks where its unique perspective enhances the understanding of node importance and network dynamics.

The proposed metric has been comprehensively evaluated on both small-scale and large-scale networks. Small-scale networks include examples with 5 nodes and 5 edges, as well as 6 nodes and 9 edges. Large-scale networks encompass real-world datasets, such as two Amazon datasets (262,111 nodes and 1,234,877 edges; 403,394 nodes and 3,387,388 edges), the Bitcoin cryptocurrency network (5,881 nodes and 35,592 edges), and a Protein–Protein Interaction (PPI) network (2,361 nodes and 7,182 edges). These evaluations demonstrate the metric’s effectiveness in identifying node importance while maintaining computational efficiency, highlighting its adaptability and relevance for analyzing networks of diverse sizes and domains.

Dangling Centrality plays a critical role in proactive decision-making, allowing planners to implement strategies that preserve system stability, even when key elements are temporarily missing. By identifying vulnerable points, this metric supports preemptive measures to maintain the network’s integrity during disruptions.

## Future directions

Future dimensions and studies for Dangling Centrality include its application to time-dependent communications, such as dynamic networks, where the network structure and node interactions evolve over time. This would provide deeper insights into the resilience of networks in changing environments. Additionally, Dangling Centrality can be extended to evaluate weighted network graphs, where the strength of connections between nodes is taken into account, allowing for more nuanced analysis of node importance based on both connectivity and weight. These extensions would enhance the versatility of Dangling Centrality, making it applicable to a broader range of real-world scenarios.

This^48^ study effectively demonstrates how fuzzy logic and centrality measures can enhance link prediction in online social networks by revealing hidden structural patterns. As an extension, *Dangling Centrality* can be applied to detect nodes with low immediate influence but high potential for future connectivity. This can provide valuable insights for predicting emerging links, especially in dynamic or partially observed network applications in^49^.

Similarly, the study like in^50^, future research can investigate the role of dangling centrality in identifying less obvious yet strategically important nodes for influence maximization. Integrating this concept with our proposed framework may enhance the detection of hidden influencers, particularly in multilayer or dynamic social networks.

## Data Availability

Data is cited in the manuscript and extracted from [http://snap.stanford.edu/data/](http:/snap.stanford.edu/data).

## Footnotes

1 ^1^https://snap.stanford.edu/data/
